# RETRACTED: Wee et al. Triaging Medical Referrals Based on Clinical Prioritisation Criteria Using Machine Learning Techniques. *Int. J. Environ. Res. Public Health* 2022, *19*, 7384

**DOI:** 10.3390/ijerph22050700

**Published:** 2025-04-29

**Authors:** Chee Keong Wee, Xujuan Zhou, Ruiliang Sun, Raj Gururajan, Xiaohui Tao, Yuefeng Li, Nathan Wee

**Affiliations:** 1 School of Business, University of Southern Queensland, Toowoomba, QLD 4350, Australia; ckwee@outlook.com (C.K.W.); raj.gururajan@usq.edu.au (R.G.); 2 Digital Application Services, eHealth, Brisbane, QLD 4000, Australia; steven.sun@health.qld.gov.au; 3 School of Mathematics, Physics and Computing, University of Southern Queensland, Toowoomba, QLD 4350, Australia; xiaohui.tao@usq.edu.au; 4 School of Computer Science, Queensland University of Technology, Brisbane, QLD 4000, Australia; y2.li@qut.edu.au; 5 Faculty of Science, University of Queensland, Brisbane, QLD 4072, Australia; nwee1@outlook.com

The journal retracts and remove the article *Triaging Medical Referrals Based on Clinical Prioritisation Criteria Using Machine Learning Techniques* [1].

Following publication, concerns were brought to the attention of the Editorial Office relating to unauthorised use of information obtained from the Queensland Health Agency, Australia. 

Adhering to our standard procedure, an investigation was conducted by the Editorial Office and Editorial Board that confirmed that appropriate permission had not been gained to use or publish potentially sensitive personal data acquired from Queensland Health Agency presented in this article. Following confirmation from the Queensland Health Agency, the Editorial Board has decided to retract and remove this publication [1], as per MDPI’s retraction policy (https://www.mdpi.com/ethics#_bookmark30). The title, author information, and this retraction note will remain permanently available, while the article itself has been removed.

This retraction was approved by the Editor-in-Chief of the *International Journal of Environmental Research and Public Health*.

Chee Keong Wee agrees to this retraction. The remaining authors did not provide a comment on this decision.

## References

[1] Wee C.K., Zhou X., Sun R., Gururajan R., Tao X., Li Y., Wee N. (2022). RETRACTED: Triaging Medical Referrals Based on Clinical Prioritisation Criteria Using Machine Learning Techniques. Int. J. Environ. Res. Public Health.

